# The macroscopic shape of assemblies formed from microparticles based on host–guest interaction dependent on the guest content

**DOI:** 10.1038/s41598-021-85816-z

**Published:** 2021-03-18

**Authors:** Takahiro Itami, Akihito Hashidzume, Yuri Kamon, Hiroyasu Yamaguchi, Akira Harada

**Affiliations:** 1 Graduate School of Science, Osaka University, 1-1 Machikaneyama-cho, Toyonaka, Osaka 560-0043 Japan; 2 The Institute of Scientific and Industrial Research, Osaka University, 8-1 Mihogaoka, Ibaraki, Osaka 567-0047 Japan

**Keywords:** Soft materials, Polymers

## Abstract

Biological macroscopic assemblies have inspired researchers to utilize molecular recognition to develop smart materials in these decades. Recently, macroscopic self-assemblies based on molecular recognition have been realized using millimeter-scale hydrogel pieces possessing molecular recognition moieties. During the study on macroscopic self-assembly based on molecular recognition, we noticed that the shape of assemblies might be dependent on the host–guest pair. In this study, we were thus motivated to study the macroscopic shape of assemblies formed through host–guest interaction. We modified crosslinked poly(sodium acrylate) microparticles, i.e., superabsorbent polymer (SAP) microparticles, with β-cyclodextrin (βCD) and adamantyl (Ad) residues (βCD(*x*)-SAP and Ad(*y*)-SAP microparticles, respectively, where *x* and *y* denote the mol% contents of βCD and Ad residues). Then, we studied the self-assembly behavior of βCD(*x*)-SAP and Ad(*y*)-SAP microparticles through the complexation of βCD with Ad residues. There was a threshold of the βCD content in βCD(*x*)-SAP microparticles for assembly formation between *x* = 22.3 and 26.7. On the other hand, the shape of assemblies was dependent on the Ad content, *y*; More elongated assemblies were formed at a higher *y*. This may be because, at a higher *y*, small clusters formed in an early stage can stick together even upon collisions at a single contact point to form elongated aggregates, whereas, at a smaller *y*, small clusters stick together only upon collisions at multiple contact points to give rather circular assemblies. On the basis of these observations, the shape of assembly formed from microparticles can be controlled by varying *y*.

## Introduction

Biological systems utilize macroscopic self-assemblies based on molecular recognition. In these decades, biological macroscopic assemblies have inspired researchers to utilize molecular recognition to develop smart materials^1–3^, e.g., soft actuators^4–9^ and self-healing materials^10–25^. Recently, macroscopic self-assemblies, which allow ones to see molecular recognition by naked eyes, have been realized using millimeter-scale hydrogel pieces possessing molecular recognition moieties^26–30^. Even if the difference in binding constants of molecular recognition moieties is small, the systems of mixture of gel pieces can exhibit perfect fidelity by controlling the contents of molecular recognition moieties^31–33^. During the study on macroscopic self-assembly based on molecular recognition, we noticed that the shape of assemblies might be dependent on the host–guest pair; For example, the pair of β-cyclodextrin (βCD) and adamantyl (Ad), which shows one of the highest binding constants, led to the formation of linear alternating assemblies, whereas βCD and *t*-butyl, which possesses a lower binding constant, might form planar checkered assemblies^26^. We were thus motivated to study the macroscopic shape of assemblies formed through host–guest interaction. We have chosen crosslinked poly(sodium acrylate) microparticles, i.e., superabsorbent polymer (SAP) microparticles, because of the availability and ease of modification. We have employed a combination of βCD and Ad as host and guest residues, respectively, because this combination forms stable inclusion complexes. In the present article, we describe preparation of SAP microparticles modified with βCD and Ad residues (βCD(*x*)-SAP and Ad(*y*)-SAP microparticles, respectively, where *x* and *y* denote the mol% contents of βCD and Ad residues), and the self-assembly behavior of βCD(*x*)-SAP and Ad(*y*)-SAP microparticles through the complexation of βCD with Ad residues, and discuss the effect of the contents of residues, i.e., *x* and *y*, on the self-assembly behavior.

## Results

### Preparation of superabsorbent polymer microparticles modified with β-cyclodextrin and adamantyl residues

**Figure 1:**
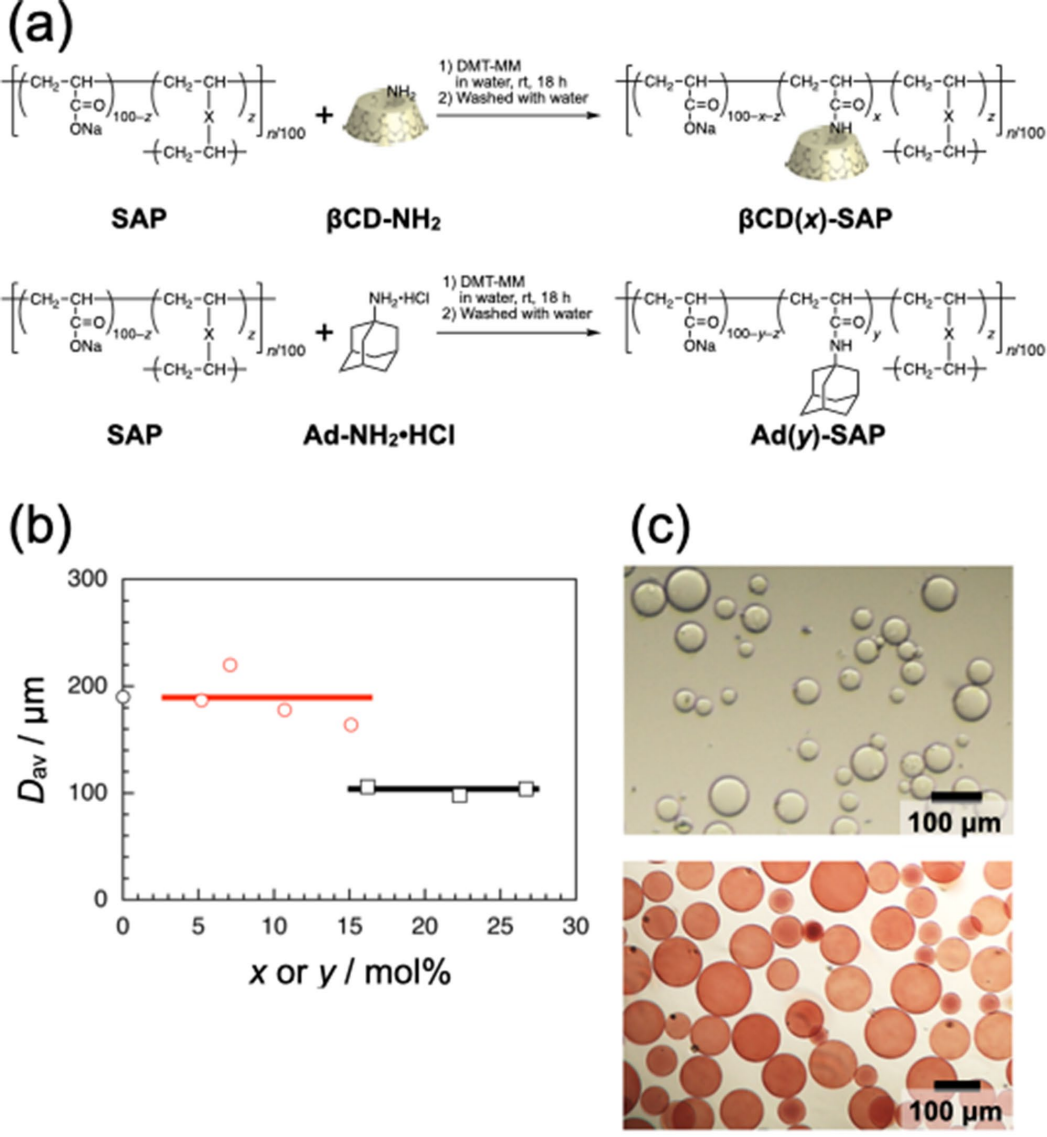
Preparation of βCD(*x*)-SAP and Ad(*y*)-SAP microparticles. (**a**) Synthetic scheme of βCD(*x*)-SAP and Ad(*y*)-SAP microparticles. (**b**) The average diameter (*D*_av_) as a function of *x* or *y* for the unmodified SAP (black circle), βCD(*x*)-SAP (black square) and Ad(*y*)-SAP microparticles (red circle). (**c**) A typical example of optical micrographs for βCD(26.7)-SAP (colorless) and Ad(15.1)-SAP microparticles (red).

We employed 10SH-NF SAP microparticles (Sumitomo Seika Chemicals Co., Ltd.) as a scaffold in this study, not only because the particles are rather spherical and uniform in size but also because the swollen SAP microparticles should be soft enough for interaction. The SAP microparticles were modified with β-cyclodextrin (βCD) and adamantyl (Ad) residues (βCD(*x*)-SAP and Ad(*y*)-SAP microparticles, respectively, where *x* and *y* denote the mol% contents of βCD and Ad residues) by coupling of mono-(6-amino-6-deoxy)-β-CD (βCD-NH_2_) and 1-adamantanamine hydrochloride (Ad-NH_2_·HCl) with carboxylic acid residues in SAP microparticles using 4-(4,6-dimethoxy-1,3,5-triazin-2-yl)-4-methylmorpholinium chloride *n*-hydrate (DMT-MM) as a coupling agent (Fig. 1a). The βCD(*x*)-SAP and Ad(*y*)-SAP microparticles were purified by washing repeatedly with water for a few days. The βCD(*x*)-SAP and Ad(*y*)-SAP microparticles prepared were characterized by solid-state ^1^H field gradient magic angle spinning (FGMAS) NMR and attenuated total reflectance (ATR) FT/IR spectroscopy (Fig. S1 and S2 in Supporting Information, respectively). As can be seen in Fig. S1, the ^1^H FGMAS NMR spectra exhibited signals ascribable to βCD and Ad residues for βCD(*x*)-SAP and Ad(*y*)-SAP microparticles at 5.32 and 1.64 ppm, respectively. As shown in Fig. S2, the ATR FT/IR spectra contain absorption bands ascribable to the stretching vibration of C=O of the amide bond at ca. 1650 cm^−1^. These spectra are indicative of successful modification of SAP microparticles.

In this study, the contents of βCD and Ad residues in βCD(*x*)-SAP and Ad(*y*)-SAP microparticles, *x* and *y*, are important parameters, but it was not possible to determine the contents of βCD and Ad residues of SAP microparticles by ^1^H FGMAS NMR because of lack of quantitativity. Thus, the values of *x* and *y* were roughly estimated by the weights of remaining βCD-NH_2_ and Ad-NH_2_·HCl recovered from the reaction mixtures, as listed in Tables 1 and 2. The βCD and Ad contents in βCD(*x*)-SAP and Ad(*y*)-SAP microparticles are markedly lower than those in feed. It should be noted here that the differences in contents are much larger for the Ad(*y*)-SAP microparticles than those for βCD(*x*)-SAP microparticles. This may be caused by the difference in reactivities of βCD-NH_2_ and Ad-NH_2_·HCl. βCD-NH_2_ is an amine, whereas Ad-NH_2_·HCl is an ammonium. The hydrochloride, Ad-NH_2_·HCl, was used because of the low solubility of 1-adamantanamine in water. The pH values of reaction media were thus different for modification with βCD and Ad residues; pH = 8.1 and 6.3 for βCD and Ad residues, respectively. Since, in amide coupling reactions using DMT-MM, the 4,6-dimethoxy-1,3,5-triazine residue is attacked by a carboxylate ion, the coupling proceeds less efficiently at lower pH^34^. Therefore, the lower pH upon modification with Ad residues may be partly responsible for the reduced Ad contents in Ad(*y*)-SAP microparticles. It should be also noted that SAP microparticles exhibited different adsorption properties for βCD-NH_2_ and Ad-NH_2_·HCl. The affinities of SAP microparticles for βCD-NH_2_ and Ad-NH_2_·HCl were compared by adsorption experiments. SAP microparticles fully swollen with water (2.0 g) were added to an aqueous solution of βCD-NH_2_ or Ad-NH_2_·HCl (5.0 mM, 10 mL). After equilibration, the concentration of solute, i.e., βCD-NH_2_ or AdNH_2_·HCl, and the volumes of aqueous solution and SAP microparticles were determined. Using these data, the amounts of βCD-NH_2_ and Ad-NH_2_·HCl adsorbed were evaluated to be ca. 52 and 2.3 mg (45 and 12 μmol), respectively, per 1 g of SAP microparticles, indicating that βCD-NH_2_ is adsorbed into SAP microparticles more strongly than Ad-NH_2_·HCl. This may be because Ad-NH_2_·HCl was salted out more readily than βCD-NH_2_ because of its hydrophobicity and concentrated Na^+^ ions inside the SAP microparticles. It is thus likely that βCD residues were introduced efficiently not only to carboxylate residues on the surface of SAP microparticles but also to those inside of SAP microparticles, whereas Ad residues were introduced dominantly to carboxylate residues on the outer layer of SAP microparticles.

**Table 1:** Conditions and results of preparation of βCD(*x*)-SAP microparticles.

Feed (mol%)	Native-SAP/mg (COOH unit/mmol)	βCD-NH_2_/mg (mmol)	DMT-MM/mg (mmol)	*x* (mol%)
30	10.1 (0.179)	60.7 (0.053)	22.3 (0.081)	16.2
40	10.2 (0.180)	81.2 (0.072)	30.0 (0.107)	22.3
100	10.1 (0.179)	203.6 (0.179)	74.5 (0.270)	26.7

**Table 2:** Conditions and results of preparation of Ad(*y*)-SAP microparticles.

Feed (mol%)	Native-SAP/mg (COOH unit/mmol)	Ad-NH_2_·HCl/mg (mmol)	DMT-MM/mg (mmol)	*y* (mol%)
10	10.0 (0.178)	3.4 (0.018)	7.5 (0.027)	5.2
30	10.2 (0.180)	10.2 (0.053)	22.3 (0.081)	7.1
50	10.0 (0.180)	16.8 (0.090)	37.1 (0.13)	10.7
200	10.2 (0.180)	67.0 (0.36)	149 (0.54)	15.1

The average diameters (*D*_av_) for a hundred of βCD(*x*)-SAP and Ad(*y*)-SAP microparticles fully swollen with water were determined by observing on an optical microscope. Values of *D*_av_ were plotted in Fig. 1b against the βCD or Ad content, i.e., *x* or *y*. The *D*_av_ values (ca. 100 μm) for βCD(*x*)-SAP microparticles were significantly smaller than that for unmodified SAP microparticles, whereas the *D*_av_ values (ca. 190 μm) for Ad(*y*)-SAP microparticles were almost the same as that for SAP microparticles independent of *y*. The smaller *D*_av_ values for βCD(*x*)-SAP microparticles indicate that βCD residues in βCD(*x*)-SAP microparticles interact with each other or with carboxylate residues presumably through hydrogen bonding formation^35,36^, resulting in a reduced swelling ratio of microparticles. These observations may support the different fashions of modification for βCD and Ad residues.

For visual discrimination, dyeing of Ad(*y*)-SAP microparticles was tested using several dyes. The food pigments, which we had used for poly(acrylamide)-based gels in our previous studies^26,27,31–33^, were not useful in this study because the pigment molecules quickly came out of Ad(*y*)-SAP microparticles. The results of dyeing test indicated that pararosaniline was a useful dye presumably because of electrostatic interaction between the ammonium in pararosaniline and carboxylate residues in Ad(*y*)-SAP microparticles (Fig. 1c). Under the dyeing conditions in this study, the size distributions of Ad(*y*)-SAP microparticles were almost the same before and after dyeing (data not shown). It is thus likely that pararosaniline has no effect on the properties of Ad(*y*)-SAP microparticles and on the interaction of βCD(*x*)-SAP and Ad(*y*)-SAP microparticles. In the following, Ad(*y*)-SAP microparticles dyed with pararosaniline were used; Colorless and red particles are βCD(*x*)-SAP and Ad(*y*)-SAP microparticles, respectively.

### Interaction of βCD(*x*)-SAP and Ad(*y*)-SAP microparticles

**Figure 2:**
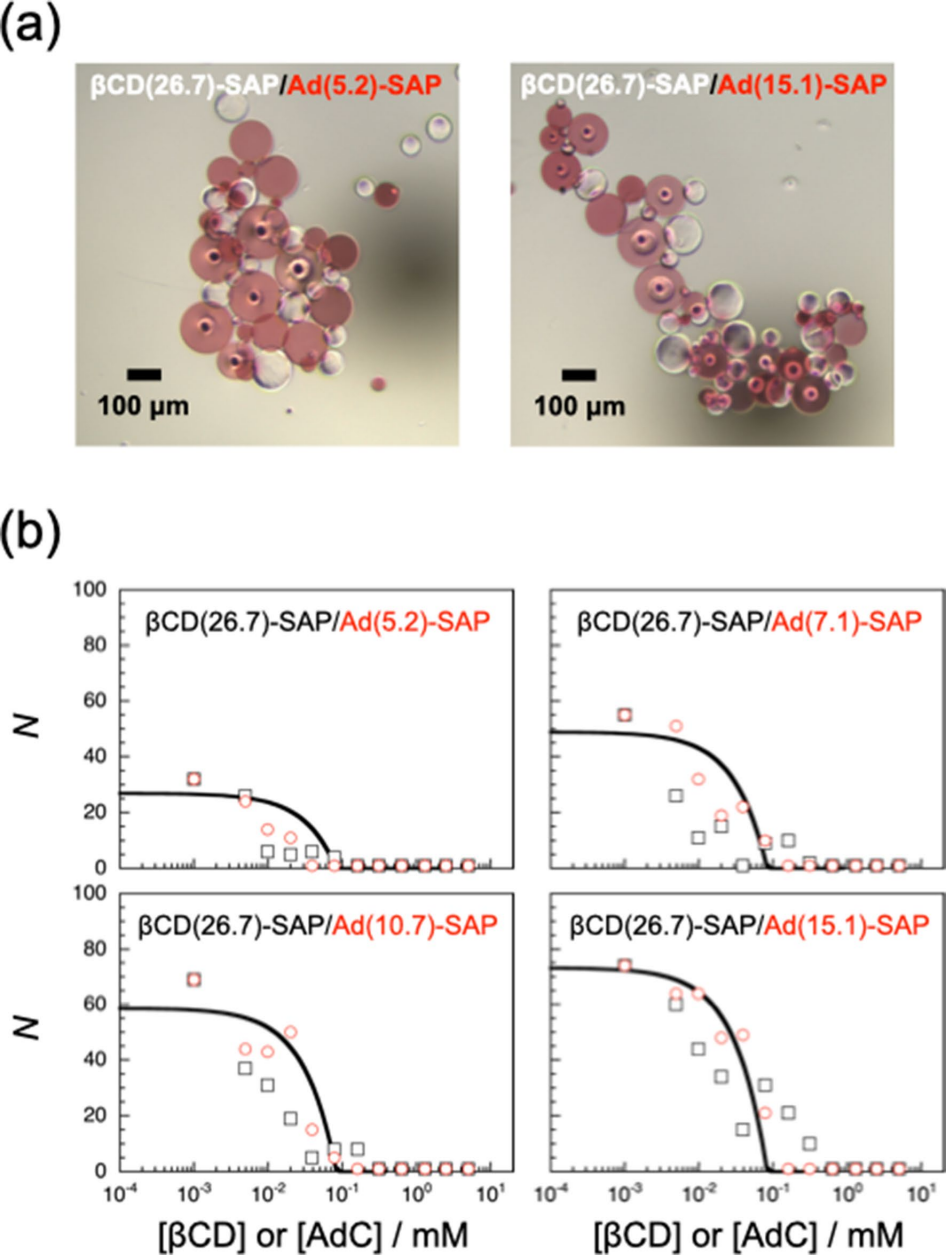
Interaction of βCD(26.7)-SAP and Ad(*y*)-SAP microparticles. (**a**) A typical example of optical micrographs for assemblies formed from βCD(26.7)-SAP and Ad(5.2)-SAP microparticles, and from βCD(26.7)-SAP and Ad(15.1)-SAP microparticles. (**b**) The number (*N*) of SAP particles in βCD(26.7)-SAP/Ad(*y*)-SAP assemblies in the presence of βCD (black square) and AdC (red circle). The curves are drawn based on the simplified model we proposed previously^37^.

The interaction of βCD(*x*)-SAP and Ad(*y*)-SAP microparticles was investigated by observing on an optical microscope. A suspension containing ca. 50 βCD(*x*)-SAP microparticles in water (1 μL) and a suspension containing ca. 50 Ad(*y*)-SAP microparticles in water (1 μL) were mixed on a clean slide glass. Since the SAP microparticles used in this study did not undergo practically Brownian motion because of their larger size, the mixed suspension was preliminarily agitated using tweezers. As can be seen in Movie S1 in Supporting Information, βCD(26.7)-SAP and Ad(15.1)-SAP microparticles adhered to each other to form an assembly. For reproducibility, the interaction studies were further carried out by agitation at 500 rpm using a mixer. Typical examples of assemblies formed are shown in Fig. 2a. Here the circles inside some microparticles were bubbles, which could not be removed. The results of interaction are summarized in Table S1 in Supporting Information. In this table, “−” denotes no assembly formation from βCD(*x*)-SAP and Ad(*y*)-SAP microparticles, and “+” denotes the formation of assemblies from microparticles. βCD(*x*)-SAP microparticles of *x* = 16.2 or 22.3 did not adhere to any of the Ad(*y*)-SAP microparticles used in this study, indicating that the number of βCD residues on the surface of βCD(*x*)-SAP microparticles of *x* = 16.2 or 22.3 was not enough for assembly formation with the Ad(*y*)-SAP microparticles. At *x* = 16.2 and 22.3, the electrostatic repulsion between carboxylate ions of βCD(*x*)-SAP and Ad(*y*)-SAP microparticles may dominate over the attractive interaction of βCD and Ad residues. On the other hand, βCD(26.7)-SAP microparticles interacted with all the Ad(*y*)-SAP microparticles examined to form assemblies. These observations indicate that there is a threshold of the βCD content in βCD(*x*)-SAP microparticles for assembly formation between *x* = 22.3 and 26.7.

The interaction of βCD(26.7)-SAP and Ad(*y*)-SAP microparticles was also investigated in the presence of competitors, i.e., βCD and sodium 1-adamantanecarboxylate (AdC). When βCD(26.7)-SAP and Ad(15.1)-SAP microparticles were agitated in water on a glass plate at 500 rpm for 1 min in the presence of 0.60 mM βCD or 0.20 mM AdC, no assemblies were formed. Since βCD molecules include Ad residues in Ad(15.1)-SAP microparticles, and AdC molecules are included in βCD residues in βCD(26.7)-SAP microparticles in these experiments, βCD or Ad residues on microparticles are masked, resulting in no assembly formation. These observations confirm that the formation of assemblies from βCD(26.7)-SAP and Ad(*y*)-SAP microparticles is based on the formation of inclusion complexes of βCD and Ad residues on the surface of SAP microparticles.

The interaction of βCD(26.7)-SAP and Ad(*y*)-SAP microparticles was also examined in the presence of varying concentrations of the competitors, i.e., βCD and AdC. In the presence of lower concentrations of the competitor, two or more assemblies were formed from fewer βCD(26.7)-SAP and Ad(*y*)-SAP microparticles. The total numbers (*N*) of microparticles forming assemblies were counted. The experiments were repeated three times under the same conditions to obtain the average *N* values. The *N* values were plotted in Fig. 2b against the competitor concentration ([βCD] or [AdC]). The dependencies of *N* on the competitor concentration are practically the same at a *y* independent of the species of competitor. As the competitor concentration increases, *N* decreases rapidly and reaches unity, i.e., no assembly formation, in the region of 10^–1^–10^0^ mM. It should be noted here that *N* values at lower competitor concentrations are larger at higher *y*. This observation indicates that the interaction of βCD(26.7)-SAP and Ad(*y*)-SAP microparticles is stronger at higher *y*^37^.

**Figure 3:**
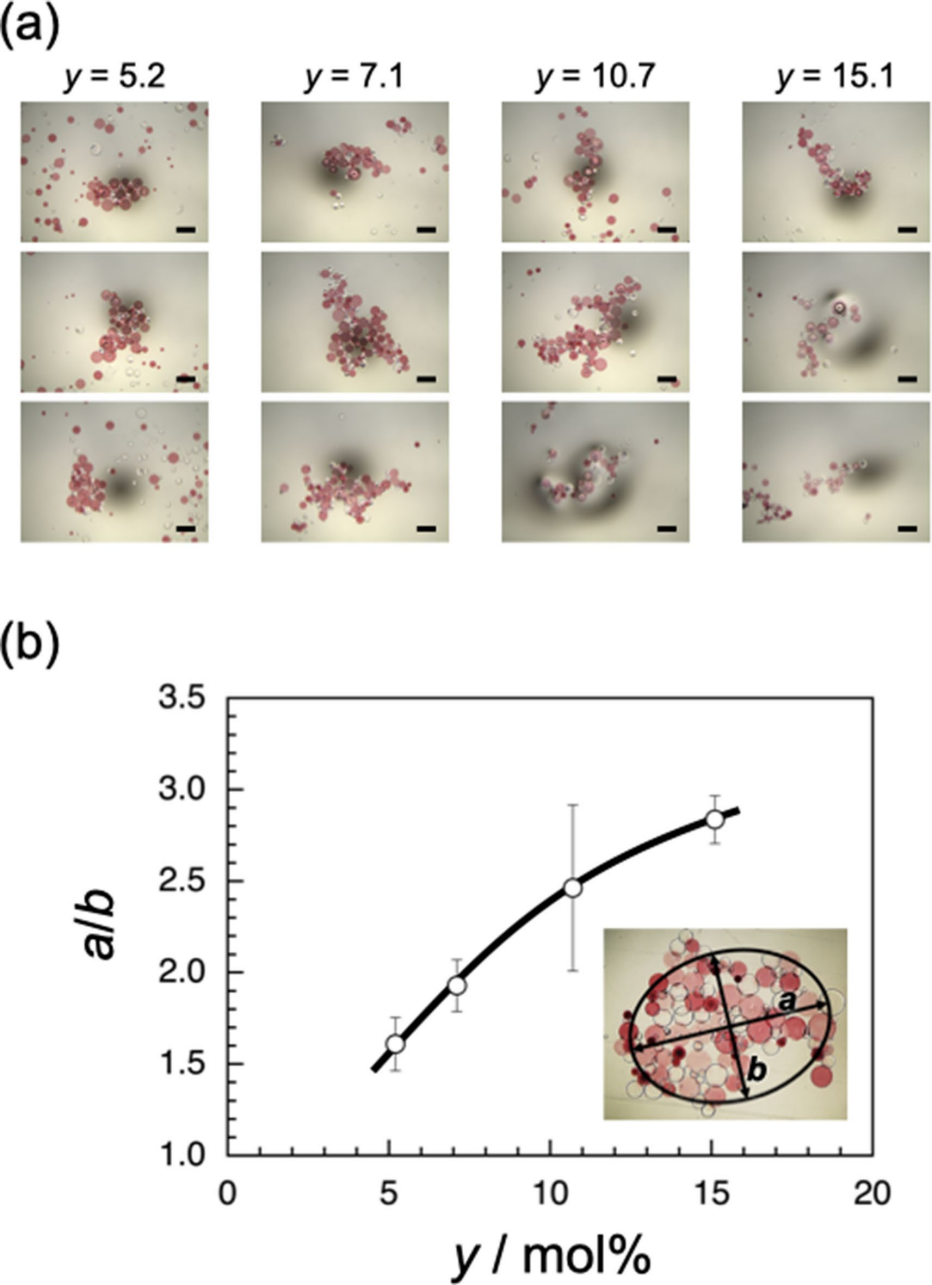
(**a**) Optical micrographs for assemblies formed from βCD(26.7)-SAP and Ad(*y*)-SAP microparticles of *y* = 5.2, 7.1, 10.7, and 15.1. The segments indicate 20 μm. (**b**) The aspect ratio, *a*/*b*, of assemblies as a function of *y*, and a typical example of estimation of *a*/*b* using an ImageJ software. The inset is an optical micrograph for the assembly formed from βCD(26.7)-SAP and Ad(5.2)-SAP microparticles.

As can be seen in Fig. 2a, the shape of assemblies seems to be dependent on the Ad content, *y*. The assembly formed at *y* = 15.1 shows a more elongated shape than does that formed at *y* = 5.2. Thus, the largest assembly formed by agitating βCD(26.7)-SAP and Ad(*y*)-SAP microparticles of each *y* at 500 rpm (Fig. 3a) was analyzed by ellipse fit using an ImageJ software^38^ to evaluate the aspect ratio (*a*/*b*, where *a* and *b* are the longer and shorter axes, respectively). The same experiments were repeated three times, and the average value of *a*/*b* was calculated. Figure 3b demonstrates the aspect ratio, *a*/*b*, as a function of the Ad content, *y*. The aspect ratio, *a*/*b*, increases from ca. 1.5 to ca. 2.8 with increasing *y* from 5.2 to 15.1. These observations indicate that assemblies of a larger aspect ratio are formed at a higher *y*, i.e., upon stronger interaction.

## Discussion

As can be seen in Fig. 3b, there is a remarkable correlation between the Ad content (*y*) in Ad(*y*)-SAP microparticles and the aspect ratio (*a*/*b*) of assemblies formed from βCD(26.7)-SAP and Ad(*y*)-SAP microparticles. In the assemblies, the interaction between βCD(26.7)-SAP and Ad(*y*)-SAP microparticles is stronger at higher *y* because of the larger number of inclusion complexes of βCD and Ad residues in a contact region. The correlation between *y* and *a*/*b* can be roughly explained as follows. Since the attractive interaction between βCD(26.7)-SAP and Ad(*y*)-SAP microparticles of a larger *y* is strong enough, small clusters formed in an early stage can stick together even upon collisions at a single contact point, resulting in the formation of elongated aggregates. On the other hand, at a smaller *y*, small clusters stick together only upon collisions at multiple contact points, leading to rather circular assemblies.

**Figure 4:**
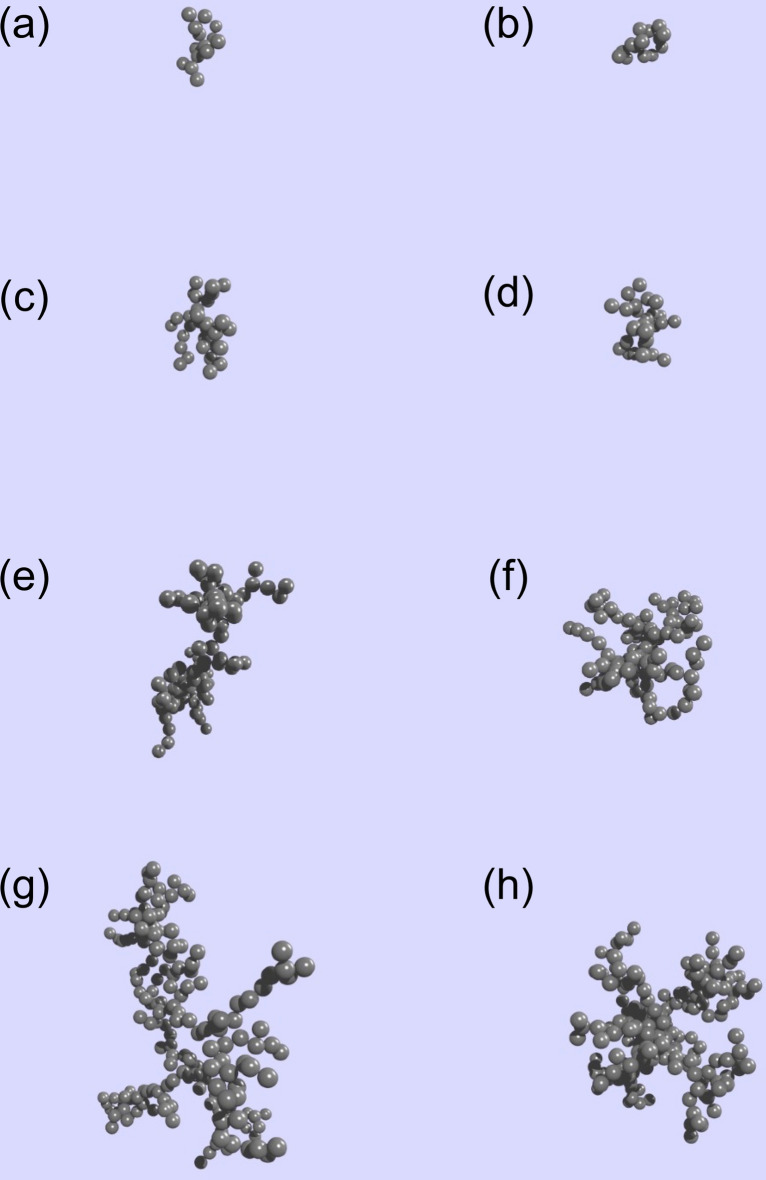
Examples for colloidal aggregates constructed using a tunable diffusion limited aggregation software^51,52^. *D*_f_ = 1.75 (**a**,**c**,**e**, and **g**) and 2.05 (**b**,**d**,**f**, and **h**) formed from 15 (**a**,**b**), 30 (**c**,**d**), 100 (**e**,**f**), and 200 particles (**g**,**h**).

Although the βCD(*x*)-SAP and Ad(*y*)-SAP microparticles are non-Brownian particles, the formation of assemblies from the microparticles is similar to aggregation of colloidal particles which undergo Brownian motion. It should be noted here that the SAP microparticles used in this study were rather spherical. Colloidal particle aggregation has been studied in detail for a century or longer^39–44^. The aggregates of colloidal particles often take fractal structures which obey

1 $$M \propto R^{{D_\text{f} }}$$

where *M* and *R* are the mass and radius of colloidal aggregate, respectively, and *D*_f_ denotes the fractal dimension^45–48^. Colloidal aggregates of larger *D*_f_ are denser, whereas aggregates of smaller *D*_f_ are less dense. On the basis of simulation and experimental studies on colloidal aggregation, it is known that *D*_f_ is ca. 1.75 for aggregates formed through the diffusion-limited aggregation and *D*_f_ is ca. 2.05 for those formed through the reaction-limited aggregation^49,50^. In the case of aggregates formed from a number of colloidal particles, the density of aggregates is dependent on *D*_f_ but the shape of aggregates is practically independent on *D*_f_. Figure 4 indicates colloidal aggregates of *D*_f_ = 1.75 (Fig. 4a,c,e and g) and 2.05 (Fig. 4b,d,f and h) formed from different numbers of particles (*N*_c_) using a Tunable Diffusion Limited Aggregation software^51,52^. As can be seen in this figure, aggregates of *D*_f_ = 1.75 are less dense than those of *D*_f_ = 2.05. It should be noted here that colloidal aggregates formed from 15 particles (*N*_c_ = 15) can be analyzed by the ellipse fit, whereas, in the cases of *N*_c_ = 30, 100, and 200, colloidal aggregates take star-shapes and may not be appropriate for the ellipse fit analysis. Using the two-dimensional projections of aggregates of *N*_c_ = 15 (Fig. 4a,b), the *a*/*b* values were estimated to be 1.75 and 1.45 for *D*_f_ = 1.75 and 2.05, respectively, with an ImageJ software. Thus, it can be concluded that the *a*/*b* ratio is dependent on *D*_f_ in the case of aggregates of smaller *N*_c_.

Since in the present system of βCD(*x*)-SAP and Ad(*y*)-SAP microparticles, assemblies are formed through the formation of inclusion complexes of βCD and Ad residues, we should also discuss binary colloidal aggregation through attractive interactions, e.g., electrostatic interaction of oppositely-charged colloidal particles^53–55^. In the case of binary colloidal aggregation, it is known that the structure of aggregates is also dependent on the ratio of radii and the ratio of the numbers of colloidal particles of different types^56–61^. In the present study, the ratio of average diameters, *D*_av_, was approximately 2 and the ratio of the numbers of particles was set to almost unity. It should be noted here that the randomness of collision is also important. In the system of βCD(*x*)-SAP and Ad(*y*)-SAP microparticles, the collisions of microparticles, i.e., non-Brownian particles, may not be purely random because the mixtures of microparticles were agitated with a mixer. On the basis of these considerations, we can conclude that the assembly of βCD(*x*)-SAP and Ad(*y*)-SAP microparticles exhibited a strong correlation between *y* and *a*/*b* because of the limited conditions, e.g., the limited number of particles, the ratios of radii and the numbers of microparticles, and the mixing procedure. It may be possible to control the structure of assemblies of βCD(*x*)-SAP and Ad(*y*)-SAP microparticles in a different manner under other conditions.

It is important to understand differences and similarities between macroscopic and microscopic assemblies. We believe that this study which compared assemblies of SAP microparticles with colloidal particles should provide significant insights.

## Conclusions

In this study, the self-assembly behavior of βCD(*x*)-SAP and Ad(*y*)-SAP microparticles was investigated. SAP microparticles were modified with βCD and Ad residues by coupling of βCD-NH_2_ and Ad-NH_2_·HCl with carboxylic acid residues in SAP microparticles using DMT-MM as a coupling agent. The contents of βCD and Ad residues in βCD(*x*)-SAP and Ad(*y*)-SAP microparticles, *x* and *y*, were roughly estimated by the weights of remaining βCD-NH_2_ and Ad-NH_2_·HCl recovered from the reaction mixtures. The adsorption experiments indicated that βCD residues were introduced efficiently not only to carboxylate residues on the surface of SAP microparticles but also to those inside of SAP microparticles, whereas Ad residues were introduced dominantly to carboxylate residues on the outer layer of SAP microparticles. For visual discrimination, Ad(*y*)-SAP microparticles were dyed with pararosaniline. The interaction of βCD(*x*)-SAP and Ad(*y*)-SAP microparticles was monitored with an optical microscope using aqueous suspension containing ca. 50 microparticles in each on a clean slide glass. Since the SAP microparticles used in this study did not undergo practically Brownian motion because of their larger size, the mixed suspension was agitated at 500 rpm using a mixer to investigate the interaction. βCD(*x*)-SAP microparticles of *x* = 16.2 or 22.3 did not adhere to any of the Ad(*y*)-SAP microparticles used in this study, whereas βCD(26.7)-SAP microparticles interacted with all the Ad(*y*)-SAP microparticles examined to form assemblies. These observations indicate that there is a threshold of the βCD content in βCD(*x*)-SAP microparticles for assembly formation between *x* = 22.3 and 26.7. Competitive experiments using βCD and AdC as competitors indicated that the formation of assemblies of βCD(26.7)-SAP and Ad(*y*)-SAP microparticles was based on the inclusion complex formation between βCD and Ad residues on the surface of SAP microparticles, and the interaction of βCD(26.7)-SAP and Ad(*y*)-SAP microparticles was stronger at higher *y*. The observation with an optical microscope indicated that the shape of assemblies was dependent on the Ad content, *y*; the aspect ratio (*a*/*b*) increased from ca. 1.5 to ca. 2.8 with increasing *y* from 5.2 to 15.1. Thus, it can be concluded that the shape of the assemblies is controlled by varying the strength of interaction of βCD(26.7)-SAP and Ad(*y*)-SAP microparticles.

## Methods

### Materials

Superabsorbent polymer 10SH-NF (SAP) microparticles were kindly supplied from Sumitomo Seika Chemicals Co., Ltd. (Osaka, Japan). Ad-NH_2_·HCl was purchased from Tokyo Chemical Industry Co., Ltd. (Tokyo, Japan). DMT-MM and D_2_O were purchased from FUJIFILM Wako Pure Chemical Corp. (Osaka, Japan). βCD was purchased from Junsei Chemical Co., Ltd. (Tokyo, Japan). Pararosaniline was purchased from Nacalai Tesque, Inc. (Kyoto, Japan). βCD-NH_2_ was prepared by previously reported procedure with slight modification^62^. Other reagents were used without further purification.

### Measurements

^1^H NMR spectra were recorded on a JEOL JNM ECA500 spectrometer. Chemical shifts were referenced to the solvent values (2.49 and 4.79 ppm for DMSO-*d*_6_ and D_2_O, respectively). Solid-state ^1^H FGMAS NMR spectra were obtained for the SAP microparticles swollen with D_2_O on a JEOL ECA400 spectrometer. Sample spinning rate was 7 kHz. ATR FT/IR spectra were measured on a JASCO FT/IR-6100 spectrometer equipped with a diamond ATR accessory. The FT/IR spectrometer was constantly purged with N_2_ gas.

### Preparation of βCD(*x*)-SAP and Ad(*y*)-SAP microparticles

βCD(*x*)-SAP and Ad(*y*)-SAP microparticles were prepared by amide coupling of carboxylate residues in the SAP microparticles with βCD-NH_2_ and Ad-NH_2_·HCl, respectively, using DMT-MM as a coupling agent in water. Predetermined amounts of SAP microparticles, βCD-NH_2_ (or Ad-NH_2_·HCl) and water were placed in a reaction vessel. After cooling the mixture at 5 °C, an aqueous solution (10 mL) of a predetermined amount of DMT-MM was added dropwise to the mixture. After agitating for 18 h at room temperature, the SAP microparticles were recovered by filtration with a glass filter, and the SAP microparticles were thoroughly washed with water to remove impurities soluble in water, i.e., unreacted DMT-MM and βCD-NH_2_ (or Ad-NH_2_·HCl) and by-products from DMT-MM. All the washings were collected and combined. After evaporation of the solvent, the residue was measured by ^1^H NMR to determine the molar ratios of components. Using the molar ratios of components and the weight of residue, the content of βCD (or Ad) residue, i.e., *x* (or *y*), was roughly estimated (Tables 1 and 2). Portions of the βCD(*x*)-SAP and Ad(*y*)-SAP microparticles obtained were lyophilized for ^1^H FGMAS NMR and ATR FT/IR spectroscopy.

For visual discrimination, the Ad(*y*)-SAP microparticles obtained were dyed by immersing in an aqueous solution of pararosaniline (0.05 M) for 1 h.

### Adsorption tests

SAP microparticles fully swollen with water (2.0 g) were added to an aqueous solution of βCD-NH_2_ or Ad-NH_2_·HCl (5.0 mM, 20 mL) and the mixture was stirred at room temperature (ca. 25 °C) for 18 h. After removing the SAP microparticles by filtration, the concentration of solution of βCD-NH_2_ or Ad-NH_2_·HCl was determined by an Anton-Paar DMA5000 density meter using the calibration curve prepared separately. Using the volume and concentration of solution, the amount of βCD-NH_2_ or Ad-NH_2_·HCl adsorbed per gram of SAP microparticles swollen was estimated.

### Interaction of βCD(*x*)-SAP and Ad(*y*)-SAP microparticles

Suspensions of βCD(*x*)SAP and Ad(*y*)SAP microparticles (1 μL each) were placed and mixed on a glass plate. The mixture was agitated at ca. 500 rpm using an EYELA CM-1000 cute mixer. The assemblies formed were observed on an EVOS optical microscope.

## Supplementary Information

Supplementary Information 1.

Supplementary Video 1.
