# Supplementary material for: The macroscopic shape of assemblies formed from microparticles based on host–guest interaction dependent on the guest content
Source: Sci Rep. 2021 Mar 18;11:6320. doi: 10.1038/s41598-021-85816-z (PMC7973530; doi:10.1038/s41598-021-85816-z)
Supplement: Supplementary file 1 — Supplementary Information 1. [file 41598_2021_85816_MOESM1_ESM.pdf]

Supporting Information for

The macroscopic shape of assemblies formed from microparticles based on host–guest interaction dependent on the guest content

Takahiro Itami[1], Akihito Hashidzume[1]*, Yuri Kamon[1], Hiroyasu Yamaguchi[1], and Akira Harada[2]*

[1]Graduate School of Science, Osaka University, 1-1 Machikaneyama-cho, Toyonaka, Osaka 560-0043, Japan

[2]The Institute of Scientific and Industrial Research, Osaka University, 8-1 Mihogaoka, Ibaraki, Osaka 567-0047, Japan

*Corresponding author.

E-mail address: hashidzume@chem.sci.osaka-u.ac.jp (AHashidzume), harada@chem.sci.osaka-u.ac.jp (AHarada).

Table S1. Results of the interaction of βCD(*x*)-SAP with Ad(*y*)-SAP microparticles[a].

	y / mol%			
x / mol%	5.2	7.1	10.7	15.1
16.2	–	–	–	–
22.3	–	–	–	–
26.7	+	+	+	+

a. "+" and "–" denote assembly formation and no assembly formation, respectively.

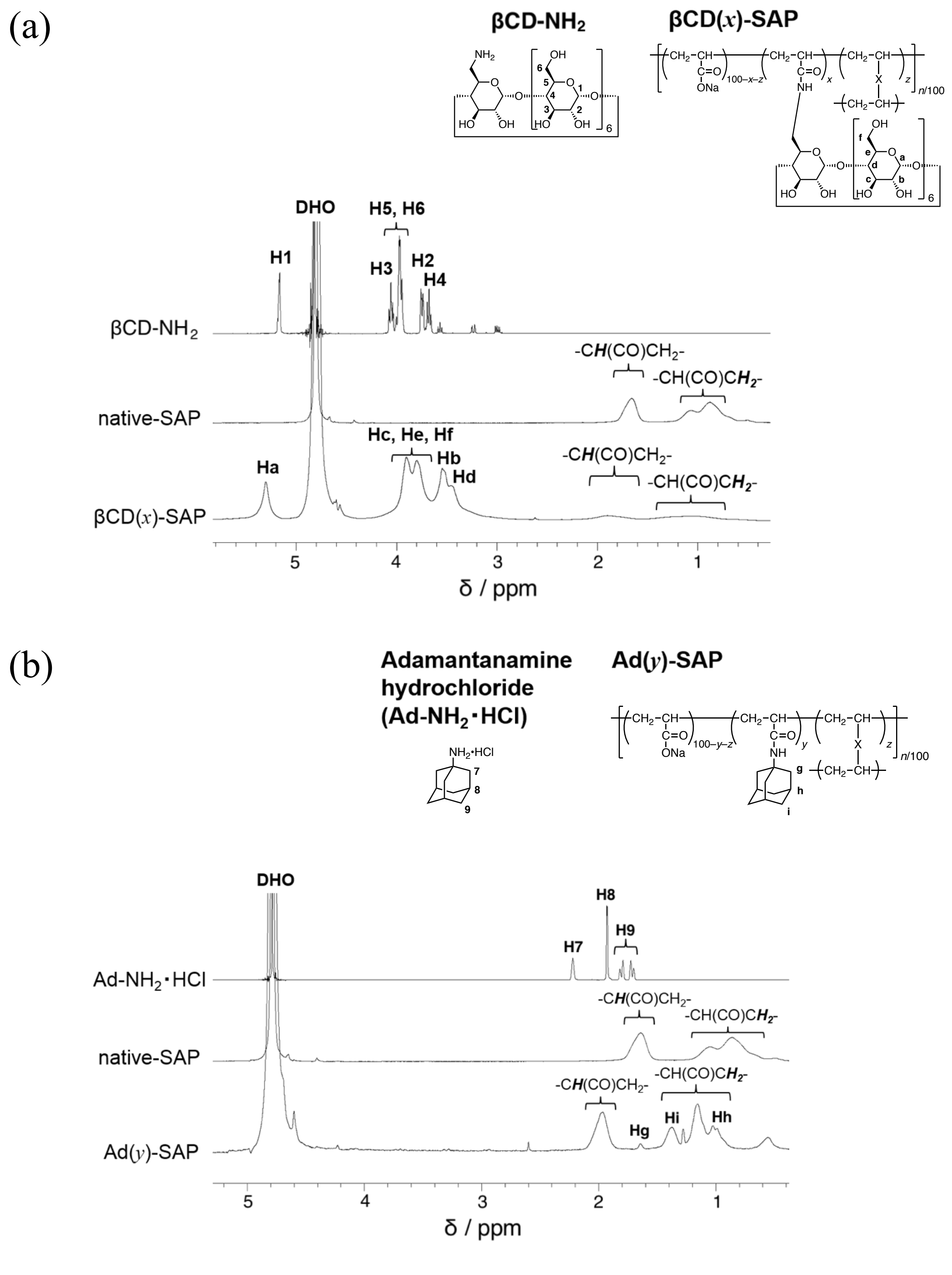

Figure S1. ^{1}H FGMAS NMR spectra for βCD-NH$_2$, Ad-NH$_2$•HCl, unmodified SAP, βCD(x)-SAP, and Ad(y)-SAP. The SAP microparticles were swollen with D$_2$O.

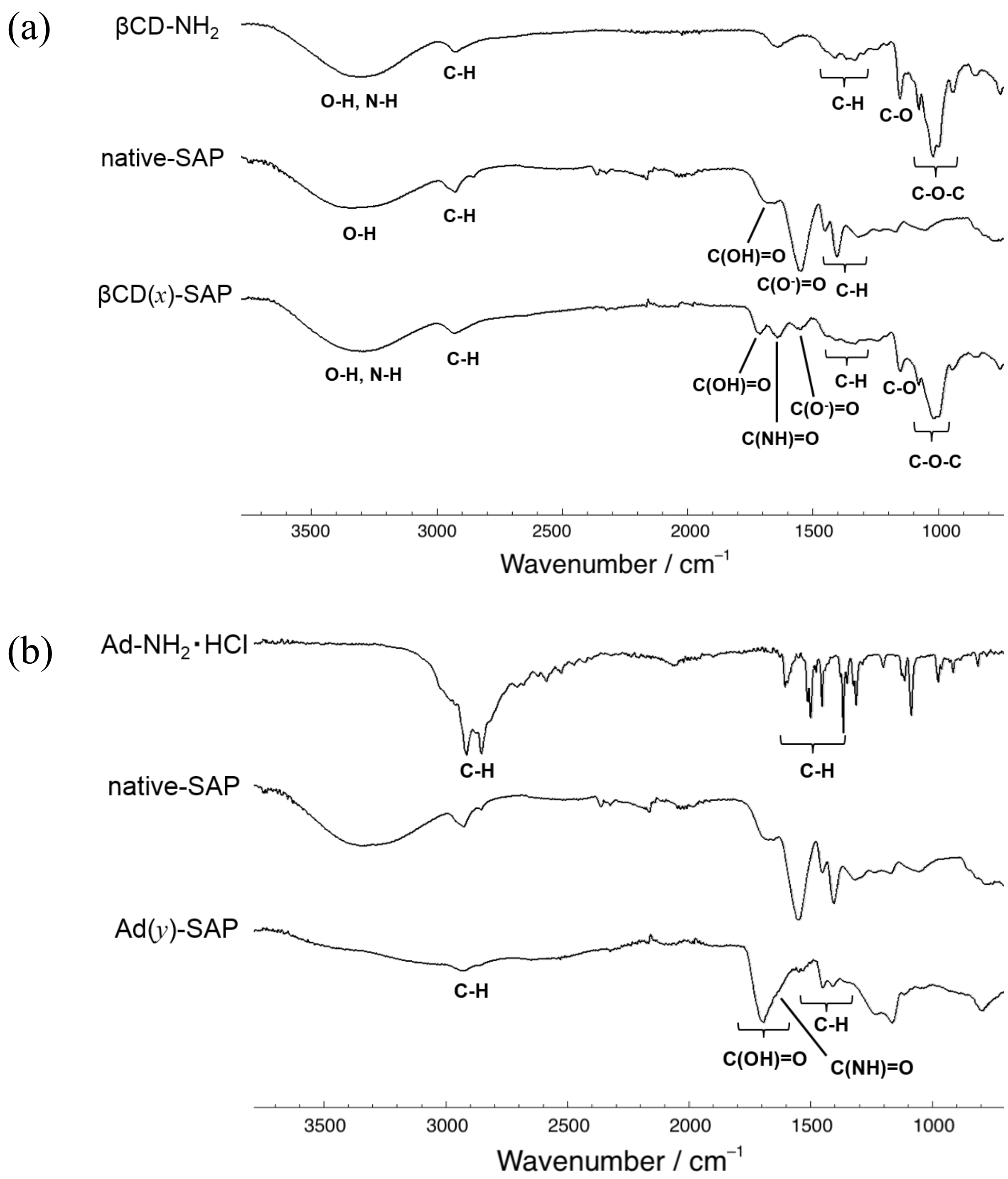

Figure S2. ATR FT/IR spectra for βCD-NH_2, Ad-NH_2•HCl, unmodified SAP, βCD(*x*)-SAP, and Ad(*y*)-SAP.
